# Novel *HSPB1* mutation causes both motor neuronopathy and distal myopathy

**DOI:** 10.1212/NXG.0000000000000110

**Published:** 2016-10-31

**Authors:** D.J. Lewis-Smith, J. Duff, A. Pyle, H. Griffin, T. Polvikoski, D. Birchall, R. Horvath, P.F. Chinnery

**Affiliations:** From the Institute of Genetic Medicine (D.J.L.-S., J.D., A.P., H.G., R.H., P.F.C.), Institute of Neuroscience (T.P.), Newcastle University; Newcastle upon Tyne Hospitals NHS Foundation Trust (D.J.L.-S., T.P., D.B., R.H.); MRC-Mitochondrial Biology Unit (P.F.C.), Cambridge Biomedical Campus; and Department of Clinical Neurosciences (P.F.C.), Cambridge Biomedical Campus, University of Cambridge, UK.

## Abstract

**Objective:**

To identify the cause of isolated distal weakness in a family with both neuropathic and myopathic features on EMG and muscle histology.

**Methods:**

Case study with exome sequencing in 2 affected individuals, bioinformatic prioritization of genetic variants, and segregation analysis of the likely causal mutation. Functional studies included Western blot analysis of the candidate protein before and after heat shock treatment of primary skin fibroblasts.

**Results:**

A novel *HSPB1* variant (c.387C>G, p.Asp129Glu) segregated with the phenotype and was predicted to alter the conserved α-crystallin domain common to small heat shock proteins. At baseline, there was no difference in HSPB1 protein levels nor its binding partner αB-crystallin. Heat shock treatment increased HSPB1 protein levels in both patient-derived and control fibroblasts, but the associated increase in αB-crystallin expression was greater in patient-derived than control fibroblasts.

**Conclusions:**

The *HSPB1* variant (c.387C>G, p.Asp129Glu) is the likely cause of distal neuromyopathy in this pedigree with pathogenic effects mediated through binding to its partner heat shock protein αB-crystallin. Mutations in *HSBP1* classically cause a motor axonopathy, but this family shows that the distal weakness can be both myopathic and neuropathic. The traditional clinical classification of distal weakness into “myopathic” or “neuropathic” forms may be misleading in some instances, and future treatments need to address the pathology in both tissues.

In the absence of other phenotypic features, the differential diagnosis of distal lower limb weakness with atrophy can be challenging. Clinical investigations are used to distinguish primary distal myopathies from a pure neurogenic disorder in an attempt to narrow down the differential diagnosis for targeted genetic analysis. Distal hereditary motor neuropathies are genetically heterogeneous and often overlapping with axonal forms of Charcot-Marie-Tooth disease, adding further complexity to the diagnostic approach. Here, we describe a family with late-onset weakness of the distal lower limbs. EMG and pathologic analysis showed evidence of both primary myopathy and motor neuropathy. Exome sequencing identified a novel mutation in the conserved α-crystallin domain of *HSPB1* affecting expression levels of its binding partner αB-crystallin following heat shock. The multiple tissues involved in this disorder are important for both the clinical classification of distal weakness and the development of new treatments.

## CASE REPORT

**Figure 1:**
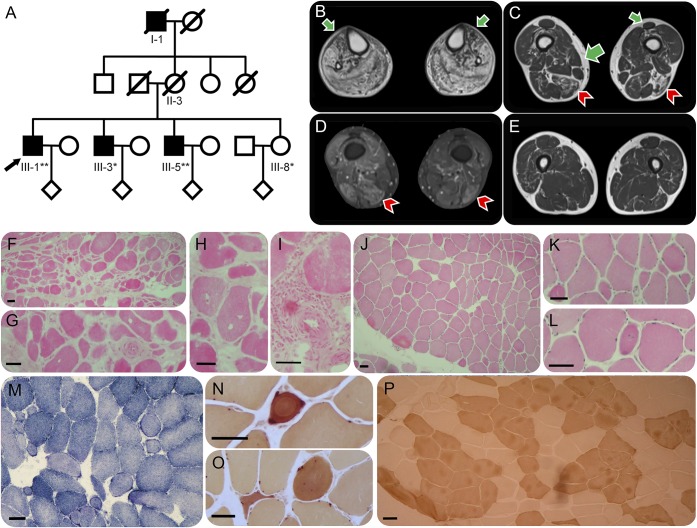
Pedigree, MRI, and muscle histology (A) Pedigree of a family of Irish descent with evidence of distal neuromuscular disease in I-1, III-1, III-3, and III-5. *DNA was available for Sanger sequencing, **DNA was available for whole-exome and Sanger sequencing. (B) T1 MRI of III-1 demonstrating severe fatty replacement of the muscles of the lower leg with relative sparing of tibialis anterior (green arrows). (C) Less marked fatty replacement and (D) active high short tau inversion recovery signal abnormality of the muscles of the lower thigh, principally affecting biceps femoris and semimembranosus (red arrowheads) but also the vasti, with relative sparing of sartorius and semitendinosus, and complete sparing of the adductors, rectus femoris, and gracilis (green arrows). (E) Minor T1 changes in the upper thigh. Muscle histology of gastrocnemius (III-1) (F–I) and of tibialis anterior (III-5) (J–P). (F) Hematoxylin & eosin (HE) staining demonstrating a wide range of fiber diameters and groups of small fibers, internal nucleation (G), and an increase in endomysial and perimysial fibrous connective tissue. (H) Scattered fibers contain vacuoles without a basophilic rim. Few fibers show acute necrosis or phagocytosis, but there is a small mononuclear cell inflammatory focus around a blood vessel (I). (J) HE staining demonstrating variation in fiber size with round atrophic fibers (K) and a slightly increased frequency of muscle fibers with internal nuclei (L). (M) Oxidative staining with NADH shows mildly moth-eaten fibers and mild subsarcolemmal accentuation in ring-shaped fibers. (N and O) Esterase histochemistry within the inset demonstrates angulated and rounded atrophic fibers with intense esterase staining and internal nuclei. (P) Adenosine triphosphatase (pH 4.3) staining demonstrating fiber-type grouping and some angulated fibers. Gastrocnemius is recognized to be more prone to secondary myopathic change than tibialis anterior. The following antibodies were used for muscle histochemistry: dystrophin: Dy10/12B2 (N term), Dy4/6 D3 (rod), and Dy8/6C5 (C term); associated glycoproteins: Ad1/20A6 (α-sarcoglycan), βSarc1/5B1 (β-sarcoglycan), 35DAG/21B5 (γ-sarcoglycan), δSarc3/12C1 (δ-sarcoglycan), and 43DAG/8D5 (β-dystroglycan); β-spectrin: RBC2/3D5 (to monitor membrane integrity on sections); laminins: commercial anti–laminin α5 (Chemicon MAB 1924), β1 (Chemicon MAB 1921) and γ1 chain (Chemicon MAB 1920), and Mer3/22B2 (equivalent to 300 kDa α2 chain fragment); caveolin 3, Emerin, and lamin A/C: commercial antibodies (Transduction Labs C38320 and Novocastra NCL-LAM-A/C, respectively); calpain 3: Calp3d/2C4 (exon 1) and Calp3c/12A2 (exon 8); dysferlin: NCL-hamlet (exon 53) and Ham3/17B2 (at exon 11-12 junction); and telethonin (G-11), neonatal myosin heavy chain (NCL-MHCn).

A 45-year-old man (III-1 in figure 1A) presented with difficulty descending stairs and instability when standing unsupported. He dragged his right foot and could not push off either forefoot. He had pes cavus, wasting of all muscles below the knee, and a bilateral high steppage gait. He was unable to stand on tiptoe or balance on either leg. Plantar flexion was weak (Medical Research Council grade 3), but the only ankle movement to overcome gravity. Ankle jerks were absent with muted plantar responses but normal sensation. Routine examination findings above the knee were normal. Serum creatine kinase (CK) was 1,060 IU/L. Other routine neuromuscular investigations were normal. Now 66 years old, the examination findings are unchanged and CK has normalized (<300 IU/L). He can walk 2 miles with orthotic aids.

III-5 presented aged 56 years with bilateral calf fasciculations and intrinsic foot muscle wasting. He was unable to spread his toes. Otherwise, examination and CK were normal. Now 64 years old, his calf and peroneal muscles remain strong and he enjoys aerobic exercise.

A further brother (III-3) developed foot drop in his fifth decade. A sister (III-8) in her sixth decade remains asymptomatic. The maternal grandfather (I-1) had foot drop and walked unsteadily by lifting his feet high and audibly slapping them on the ground. The mother (II-2) remained independently ambulant with occasional cramps into her eighth decade.

Initial neurophysiologic examination of III-1 and III-5 demonstrated reduced distal compound muscle action potentials with normal conduction velocities, but with greater jitter in tibialis anterior than attributable to denervation. Ten years later, III-1 had typical myopathic motor unit potentials in the legs, slowing of motor and sensory conduction and loss of distal sensory nerve action potentials (table e-1 at Neurology.org/ng).

Serial MRI of III-1 demonstrated early severe fatty infiltration of all lower leg muscles with relative sparing of tibialis anterior (figure 1B). Subsequently, selective fatty replacement and edema affected the thigh muscles (figure 1, C and D), with severity of involvement increasing with distance from the hips (figure 1, C and E).

Histologic examination of gastrocnemius in III-1 suggested a severe myopathy (figure 1, F–I), with normal immunohistochemistry (table e-2), immunoblot, and mitochondrial complex function. Tibialis anterior of III-5 showed histologic evidence of both myopathic and neurogenic changes (figure 1, J–P).

The predominantly myopathic phenotype led to candidate gene analysis of known distal myopathy genes *DES*, *CRYAB* (encoding αB-crystallin), *MYOT*, *LBD3*, *TTN*, *VCP*, *FKRP*, and *GNE*, which were normal.

## LABORATORY METHODS AND RESULTS

Illumina TruSeq 62 Mb exome capture sequencing (100 bp paired-end reads, HiSeq 2000) and alignment (UCSC hg19) was performed on DNA from III-1 and III-5. No genetic variants known to cause similar phenotypes were found (coverage data in table e-3). Variants with minor allele frequency >0.01 in local and international databases were discarded. Heterozygous and X-chromosome variants and those affecting genes implicated in neuromuscular diseases were prioritized bioinformatically (tables e-4 through e-8).

**Figure 2:**
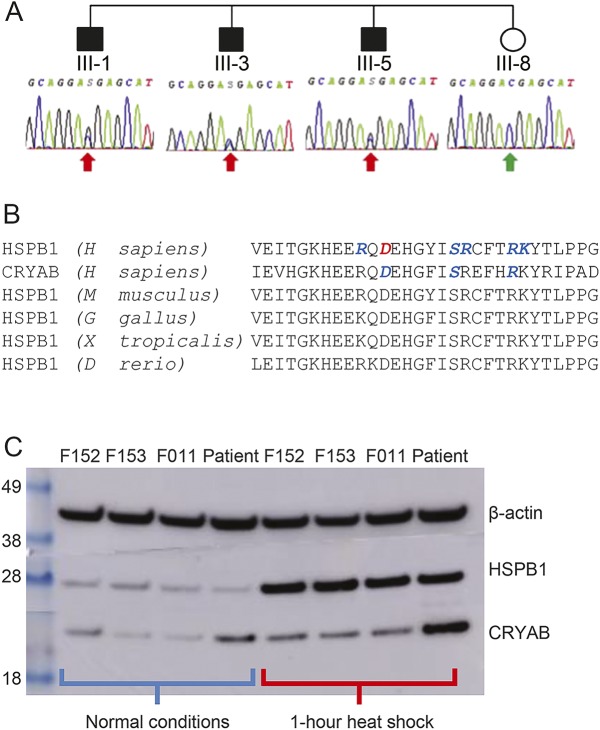
Sequence validation, functional conservation, and Western blot data (A) Sanger sequencing electropherograms demonstrating the segregation of the heterozygous *HSPB1* c.387C>G variant with the disease phenotype in the 4 siblings. (B) Correspondence of the novel pathologic α-crystallin domain mutation site (in red) in human HSPB1 to human CRYAB and HSPB1 analogs in other species. Sites of known pathologic mutations are in blue. (C) Western blot of HSPB1 and CRYAB expression in control (F152, F153, and F011) and patient-derived fibroblasts before, and immediately after, heat shock by 1-hour incubation at 44°C.

A novel missense mutation in *HSPB1* (c.387C>G, p.Asp129Glu) was confirmed in all 3 affected brothers (III-1, III-3, and III-5) by Sanger sequencing (table e-9 and figure e-1) and absent in the clinically unaffected sister, III-8 (figure 2A). This mutation lies within the characteristic, and highly conserved α-crystallin domain of small heat shock proteins, often implicated by *HSPB1*- and *CRYAB*-related diseases (figure 2B).

Expression of *HSPB1* and *CRYAB* was increased following 1 hour of heat shock by incubation at 44°C in both control- and patient-derived fibroblasts (figure 2C). At baseline and after heat shock, there was no difference in *HSPB1* expression between control- and patient-derived fibroblasts. However, while patient and control *CRYAB* expressions were indistinguishable at baseline, *CRYAB* expression after heat shock was greater in patient-derived fibroblasts than that in controls.

### Standard protocol approvals, registrations, and patient consents.

This study had national ethical approval and institutional review board approval, and patients provided informed consent.

## DISCUSSION

Eighteen pathogenic mutations in *HSPB1* have been previously described in patients with a motor axonal neuropathy (collated in reference 1, original articles cited in the supplementary references), either in isolation as a distal hereditary motor neuropathy or as Charcot-Marie-Tooth 2F in the presence of clinical sensory neuron involvement. Although the phenotype seen in the patients we described bears some resemblance to the previously published cases, the myopathic features are unusual. This is important because initial clinical presentation in our family, with CK over 1,000 IU/L and muscle histology indicated a predominantly myopathic process, pointed toward a genetically determined distal myopathy, and the differential diagnosis did not include mutations in *HSPB1*. Targeted sequencing of genes previously known to cause a distal myopathy did not identify the underlying cause, which was only revealed through whole-exome sequencing.

The *HSPB1* (c.387C>G) variant is highly likely to be responsible for the disorder in the family we describe here because (1) it was not present in healthy controls (not present in Exome Aggregation Consortium or Single Nucleotide Polymorphism Database); (2) it segregated with the phenotype; (3) it is predicted to disrupt a highly conserved amino acid residue within the α-crystallin domain of HSBP1 (p.Asp129Glu), a region of the protein containing other pathogenic mutations; and (4) the variant increased *CRYAB* expression in response to heat shock.

HSPB1 and αB-crystallin are both stress-inducible, adenosine triphosphate–independent small heat shock proteins expressed in skeletal muscle and neurons, which interact to form large hetero-oligomers. HSPB1 enhances the stability of αB-crystallin,^2^ which is the better chaperone.^3^ Heterozygous αB-crystallin p.Asp109His,^4^ p.Arg120Gly,^5^ and other^6,7^ mutations have been associated with the accumulation of αB-crystallin in patients with similar distal myopathic features to the family we describe here. It is also interesting that a similar pattern of muscle and nerve involvement was recently reported in 2 families with distal weakness caused by mutation in the related protein encoded by *HSPB8*.^8^

Disruption of the interaction between Asp109 and Arg120 during αB-crystallin homodimerization is predicted to be important in causing a myopathy.^4^ The HSPB1 p.Asp129Glu and αB-crystallin p.Asp109His substitutions correspond to the same α-crystallin domain aspartate residue (figure 2B). Thus, the elevated CK seen in our family may result from an alteration in the analogous aspartate-arginine interaction during HSPB1–αB-crystallin heterodimerization, leading to the accumulation of αB-crystallin on heat shock. This pathologic model predicts that primary myopathic features may also be present in individuals with the HSPB1 p.Arg140Gly substitution,^9^ equivalent to αB-crystallin p.Arg120Gly and with similar physicochemical consequences.^10^

Intriguingly, the presence of the αB-crystallin p.Asp109His variant has been shown sufficient to increase the accumulation of αB-crystallin and the rate of apoptosis in transfected HeLa cells via mechanisms mitigated by wild-type HSPB1.^11^ Should the HSPB1 p.Asp129Glu substitution compromise these roles of HSPB1, then any αB-crystallin accumulation and related apoptosis could proceed unchecked, independently of any defective heteroligomeric α-crystallin domain interaction.

In exome studies, it is tempting to overlook heterozygous mutations in genes not previously linked to the phenotype because many are rare or private polymorphisms. However, a broader consideration of the underlying disease mechanisms, and particularly the relevance of key interacting proteins, will assist in the prioritization of novel variants for further functional studies. This will help establish pathogenicity and broadening our understanding of the relationship between genotype and phenotype. A deeper knowledge of the cell types involved in widely expressed genes is critical for the development of novel therapies.

## Supplementary Material

Data Supplement

## GLOSSARY

CK: creatine kinase
