# Supplementary material for: Novel HSPB1 mutation causes both motor neuronopathy and distal myopathy
Source: Neurol Genet. 2016 Oct 31;2(6):e110. doi: 10.1212/NXG.0000000000000110 (PMC5089436; doi:10.1212/NXG.0000000000000110)
Supplement: Data Supplement [file supp_2_6_e110__index.html]

Data Supplement 

# Novel *HSPB1* mutation causes both motor neuronopathy and distal myopathy

## Data Supplement

**Files in this Data Supplement:**

- Data Supplement - PDF
