# Supplementary material for: Novel HSPB1 mutation causes both motor neuronopathy and distal myopathy
Source: Neurol Genet. 2016 Oct 31;2(6):e110. doi: 10.1212/NXG.0000000000000110 (PMC5089436; doi:10.1212/NXG.0000000000000110)
Supplement: Data Supplement [file supp_2.6.e110_Supplementary_material.pdf]

Supplementary Material

1: Clinical Data

Two brothers (III:1 and III:5) affected by an unexplained inherited distal motor phenotype were recruited from a regional neurogenetic clinic (Newcastle upon Tyne NHS Foundation Trust, UK) and investigated between 1992 and 2015. A further affected brother (III:3) and unaffected sister (III:8) were consented and consulted remotely. All 4 participants were approached and provided written consent for participation as previously agreed with our local research ethics committee. Detailed neurophysiological results are shown in Table e-1.

2: Whole-exome Sequencing and Variant Filtering

Genomic DNA was isolated from circulating lymphocytes (III:1 & III:5) and saliva (III:3 & III:8) (DNeasy, Qiagen, Valencia, CA), fragmented and enriched (Illumina TruSeq 62 Mb exome capture), and sequenced (Illumina HiSeq 2000, 100 bp paired-end reads). The in-house bioinformatics pipeline followed the standard procedure of alignment to the human reference genome (UCSC hg19),[1] removal of duplicate sequence reads using Picard v1.85,[2] and variant detection using Varscan v2.2 [3] and Dindel v1.01.[4]

Whole-exome sequencing coverage of selected genes of interest was adequate to exclude known mutations capable of causing the participants' phenotype, and supplemented with clinically validated PCR-based tests (Tables e-3 tp e-9, & Figure e-1).

Table e-1 Neurophysiological data

Individual	Age (y)	Motor												Sensory							
		Right Median			Right Ulnar			Right Common Peroneal			Right Posterior Tibial			Right Radial		Right Ulnar		Right Superficial		Right Sural	
		Amplitude (µV)	Velocity (m/s)	F latency (ms)	Amplitude (µV)	Velocity (m/s)	F latency (ms)	Amplitude (µV)	Velocity (m/s)	F latency (ms)	Amplitude (µV)	Velocity (m/s)	F latency (ms)	Amplitude (µV)	Velocity (m/s)	Amplitude (µV)	Velocity (m/s)	Amplitude (µV)	Velocity (m/s)	Amplitude (µV)	Velocity (m/s)
III:1	47	16000	59	26.2-29.4	NE	NE	NE	400	45	NR	120	34	NR	15	47	13	52	NE	NE	7	37
III:1	57	11900	60	29.2-32.5	7500	68	31.3-33.8	300	29	NE	NR	NR	NR	8	66	5	66	NR	NR	3	40
III:5	56	20600	56	32.8-34.6	11000	59	33.5-35.3	NR	NR	NR	100	42	NR	6	61	8	59	8	43	14	45

NR= Not recordable, NE= Not examined

Individual	Age (y)	Muscles Examined	Concentric Needle EMG
III:1	47	Right FDI, FCR, FDS, VM, VL, TA & MG, & left TA	Spontaneous fibrillations and positive sharp waves were recorded in right and left TA and right MG. Recruitment was reduced in density in the TA, MG & VM. An excess of irregular and polyphasic motor unit potentials of up to 7 mV amplitude and slightly unstable outline was seen in the distal muscles.
III:1	57	Right FCR, FDS, VL, TA & MG	Spontaneous fibrillation potentials and positive sharp waves in TA & MG. Needle insertion in these muscles was met resistance giving a "woody" feel consistent with fibrous replacement of muscle. Volitional activity was discrete in TA and much reduced in MG. Motor unit action potentials were of short to normal duration and reduced amplitude in TA & MG, and simple or irregular in shape and stable in outline. In VL the motor unit potentials were of slightly increased duration, 0.5 to 2.2 mV in amplitude and stable in outline. In FCR & FDS recruitment was normal with full interference patterns and motor unit potentials of 0.5 to 1.5 mV amplitude, normal duration and stable outline. Interpretation: marked muscle fibre loss and fibrous replacement in distal muscles, with changes in motor units more typical of a myopathic disorder.
III:5	56	Right FDI, FDS, EDC, VL, TA & MG	Spontaneous fibrillation potentials and positive sharp waves together with fasciculation potentials were recorded in MG. Volitional activity was reduced in density in VL, TA & MG with an excess of irregular and polyphasic motor unit potentials, some with late components of increased duration and amplitude and slightly unstable outline. Quantitative multimup analysis confirmed these findings. In EDS, FDS & FDI recruitment was normal with full interference patterns and normal, stable motor unit potentials.

Single fibre EMG performed in the 22 pairs of right tibialis anterior muscle of III:5 aged 56 years demonstrated increased jitter in 50% of pairs and blocking in 5% of pairs. Macro EMG: FD 2.3, Mean value 64 µS MCD. Macro amplitude 1181 µV, Range 323-3918 µV. Interpretation: FD and macro motor unit amplitudes increased, and quantitative MUAP analysis demonstrated increased amplitutdes and durations in keeping with chronic neurogenic process. However jitter was beyond that expected for a pure deinnervation and reinnervation process.

FDI= first dorsal interosseous, FCR= flexor carpi radialis, FDS= flexor digitorum sublimis, EDC= extensor digitorum communis, VM= vastus medialis, VL= vastus lateralis, TA= tibialis anterior, MG= medial gastrocnemius

Table e-2. Immunohistochemical labels

Immunohistochemistry targets
Dystrophin, sarcoglycans, laminins, caveolin 3, emerin, calpain 2, dysferlin

Table e-3. Exome sequencing coverage of genes with known similar phenotypes

		Participant	Bases covered (%)					Clinical Test
			1-fold	5-fold	10-fold	20-fold	30-fold	
All genes		III:1	99.8	99.5	98.8	94.7	86.4	
		III:5	99.8	99.5	98.8	95	86.5	
DES	AD/AR Myofibrillar myopathy 1 (Distal onset neuropathy with cardiomyopathy)	III:1	100	100	100	100	99.1	Excluded
		III:5	100	100	100	100	97.9	
CRYAB	AD Myofibrillar myopathy 2 (Distal myopathy with or without cataracts)	III:1	100	100	100	89.6	72.2	Excluded
		III:5	100	100	100	83.3	60.2	
MYOT	AD Myofibrillar myopathy 3 (distal leg myopathy +/- neuropathy)	III:1	88.6	78.1	76.2	71	56.2	Excluded
		III:5	91.7	79.8	75.5	61.6	53.8	
LBD3	AD Myofibrillar myopathy 4 (distal leg myopathy +/- neuropathy)	III:1	97.4	95	93.5	89.5	82.9	Excluded
		III:5	94.5	93.9	93	88.7	81.7	
FLNC	AD Myofibrillar myopathy 5 (Proximal or rarely distal leg myopathy +/- neuropathy)	III:1	99.1	98	95.1	88	78.7	
		III:5	100	97.9	92.5	85.8	77	
BAG3	AD Myofibrillar myopathy 6 (Severe adolescent-onset generalised myopathy with	III:1	100	100	100	99.5	91.4	

	cardiomyopathy +/- neuropathy)	III:5	100	100	100	97.3	89.2	
GNE	AR Nonaka myopathy (distal leg myopathy without neuropathy)	III:1	100	100	100	98.7	87	Excluded
		III:5	100	100	100	99.4	90.9	
DYSF	AR Distal myopathy with tibial onset, Miyoshi myopathy and LGMD2B	III:1	100	100	99.9	96.8	88.4	
		III:5	100	99.9	99.3	97.2	88.7	
ANO5	AR Miyoshi myopathy 3 (Adult onset distal muscular weakness)	III:1	100	100	100	92.3	79.9	
		III:5	100	100	99.8	97.5	81.5	
TTN	AD Tardive tibial muscular dystrophy (and others)	III:1	100	99.8	99.2	94.6	85	Excluded
		III:5	100	99.9	99.4	95.3	86.1	
VCP	AD Inclusion body myopathy, amyotrophic lateral sclerosis, Paget's disease, early-onset frontotemporal dementia	III:1	100	100	100	98	95.8	Excluded
		III:5	100	100	97.9	95.2	91.9	
MYH7	AD Scapuloperoneal dystrophy	III:1	100	100	99.9	98.8	97.2	
		III:5	100	99.9	99.6	98.8	93.8	
MATR3	AD Amyotrophic lateral sclerosis (with myopathic features)	III:1	100	98.1	95.9	90	76.5	
		III:5	99.2	98.1	96.7	91.7	79.5	
NEB	AR Nemaline myopathy (AR childhood onset)	III:1	100	100	99.8	97.5	89.9	
		III:5	100	99.9	99.8	97.4	90.8	

Table e-4. Filtering of whole-exome sequencing variants for III:1 and III:5

<table>
<tr><td colspan="5">Any variation from reference sequence present in either brother</td><td>99,189</td></tr>
<tr><td colspan="5">Protein-altering mutations: Non-synonymous substitutions, start codon gain or loss, stop codon gain or loss, and insertions or substitutions located in exons or splice sites</td><td>13,454</td></tr>
<tr><td colspan="3">Heterozygous variants sufficiently rare to cause rare autosomal dominant disease Minor allele frequency <0.001</td><td>234</td><td>Homozygous or X-chromosome variants sufficiently rare to cause rare recessive or X-linked disease Minor allele frequency <0.1</td><td>443</td></tr>
<tr><td colspan="3">Heterozygous in both brothers</td><td>67</td><td>Homozygous in both brothers</td><td>0</td></tr>
<tr><td rowspan="4">Consisting of</td><td rowspan="2">Substitutions</td><td>Missense</td><td>60</td><td rowspan="2">X-chromosome variants present in both brothers</td><td rowspan="2">0</td></tr>
<tr><td>Nonsense</td><td>4</td></tr>
<tr><td rowspan="2">Insertions or deletions</td><td>Non-frame shift</td><td>2</td><td colspan="2" rowspan="2"></td></tr>
<tr><td>Frame shift</td><td>1</td></tr>
</table>

Preliminary filtering selected those variants:

1) Predicted to alter resultant proteins (non-synonymous substitutions, stop loss, stop gain, frame shift and non-frame shift insertions and deletions in exons and splice sites) using Annovar,[5]
2) With minor allele frequencies sufficiently low to cause rare Mendelian disease (maximum 0.1 for homozygous and X-linked variants, and 0.001 for heterozygous variants) on cross-referencing each variant to international databases (dbSNP135,[6] 1000 genomes [7] and Exome Sequencing Project 6500 exomes)[8] and 238 unrelated controls from our local population.
3) Present in both III:1 and III:5.

Table e-5. Genes containing rare insertions, deletions & nonsense variants in both brothers (some variants involved the coding regions of multiple genes)

MAK, NUP43, SP9, SURP, TTK, XIRP1, ZNF823

Table e-6. Rare missense variants present in both brothers and predicted to have deleterious functional consequences in all models on Annovar [5]

DBN1	ENST00000309007:c.C748T:p.R250W
DOCK7	ENST00000340370:c.C3316A:p.P1106T
EAPP	ENST00000554792:c.G343A:p.E115K
GCC1	ENST00000321407:c.G1234A:p.A412T
HSPB1	ENST00000248553:c.C387G:p.D129E
KAT2A	ENST00000225916:c.C2353T:p.R785C
LRFN2	ENST00000338305:c.C2236T:p.R746W
LRRC45	ENST00000306688:c.T797C:p.I266T

ENST Transcript labels refer to the Ensembl database.[9]

Table e-7. Variants present in both brothers in genes implicated in peripheral neuromuscular disease. *HSPB1* p.D129E is not present in Exac or dbSNP; *NEFH* p.V928L is present Exac = 3.422e-05, rs199532217; and *TTN* p.L12818F is also present in Exac = 0.0001866, rs374736305.

HSPB1 ENST00000248553:c.C387G:p.D129E	AD Charcot Marie Tooth 2F & AD distal hereditary motor neuropathy IIB
NEFH ENST00000310624:c.G2782T:p.V928L	Amyotrophic lateral sclerosis (combination of long & deleted allele)
TTN ENST00000460472:c.G38454T:p.L12818F	AD tardive tibial muscular dystrophy

Table e-8. Variants of interest in genes implicated in peripheral neuromuscular disease present in one brother with inadequate coverage to exclude its presence in the other brother

NEFH ENST00000310624:c.1952_1975del:p.651_659del	Amyotrophic lateral sclerosis (combination of long & deleted allele)
PLEC ENST00000322810:c.G9484A:p.V3162I	AR limb girdle muscular dystrophy 2Q
PLEC ENST00000322810:c.G305T:p.R102L	AR limb girdle muscular dystrophy 2Q

Table e-9. Sanger sequencing primers and conditions

Polymerase chain reaction conditions

Single reaction	Volume (µl)
Immunobuffer (x10)	2.5
dNTP (2mM)	2.5
Immolase	0.2
Forward primer (10µM)	0.63
Reverse primer (10µM)	0.63
DNA (25-50ng)	1
$MgCl_2$ (50mM)	2
Betaine (5M) if required	5
Water to make up 25 µl	10.54-15.54

Thermal cycler settings

Activation: pre-heat at 95°C for 7 minutes

40 cycles of: Denaturation: 95°C for 1 minute, annealing: primer-dependent for 30 seconds & extension at 72°C for 30 seconds

Final extension at 72°C for 10 minutes

Primer sequences:

***HSPB1* ENST00000248553:c.C387G:p.D129E**

Specific conditions: $t_{annealing}$ 65°C with betaine

Forward CGTACTGCTCACTCCCCAG

Reverse TCTCATCGGATTTTGCAGCT

***NEFH* ENST00000310624:c.1952_1975del:p.651_659del**

Specific conditions: $t_{annealing}$ 65°C with betaine

Forward CCCCAGCCGAAGTCAAGT

Reverse TGGGATCTCCTTCTCAGGGG

***PLEC* ENST00000322810:c.G9484A:p.V3162I**

Specific conditions: $t_{annealing}$ 65°C

Forward GTACCCTGTCACAGCCTTCT

Reverse CTGGAGAGCAGGGTCATCG

***PLEC* ENST00000322810:c.G305T:p.R102L**

Specific conditions: $t_{annealing}$ 65°C with betaine

Forward CGACAACCAGACCCCTACTT

Reverse CCTGGTGCCACTTTTACTGG

***TTN* ENST00000460472:c.G38454T:p.L12818F**

Specific conditions: $t_{annealing}$ 58°C

Forward CCTGAGTCCTTCCGGTTCAC

Reverse AGACCTGAGTGTGGCTATG

Figure e-1. Sanger sequencing electropherogram HSPB1 *c.387C>G* region in the participating family members
The blue column heights indicate the signal quality of each base

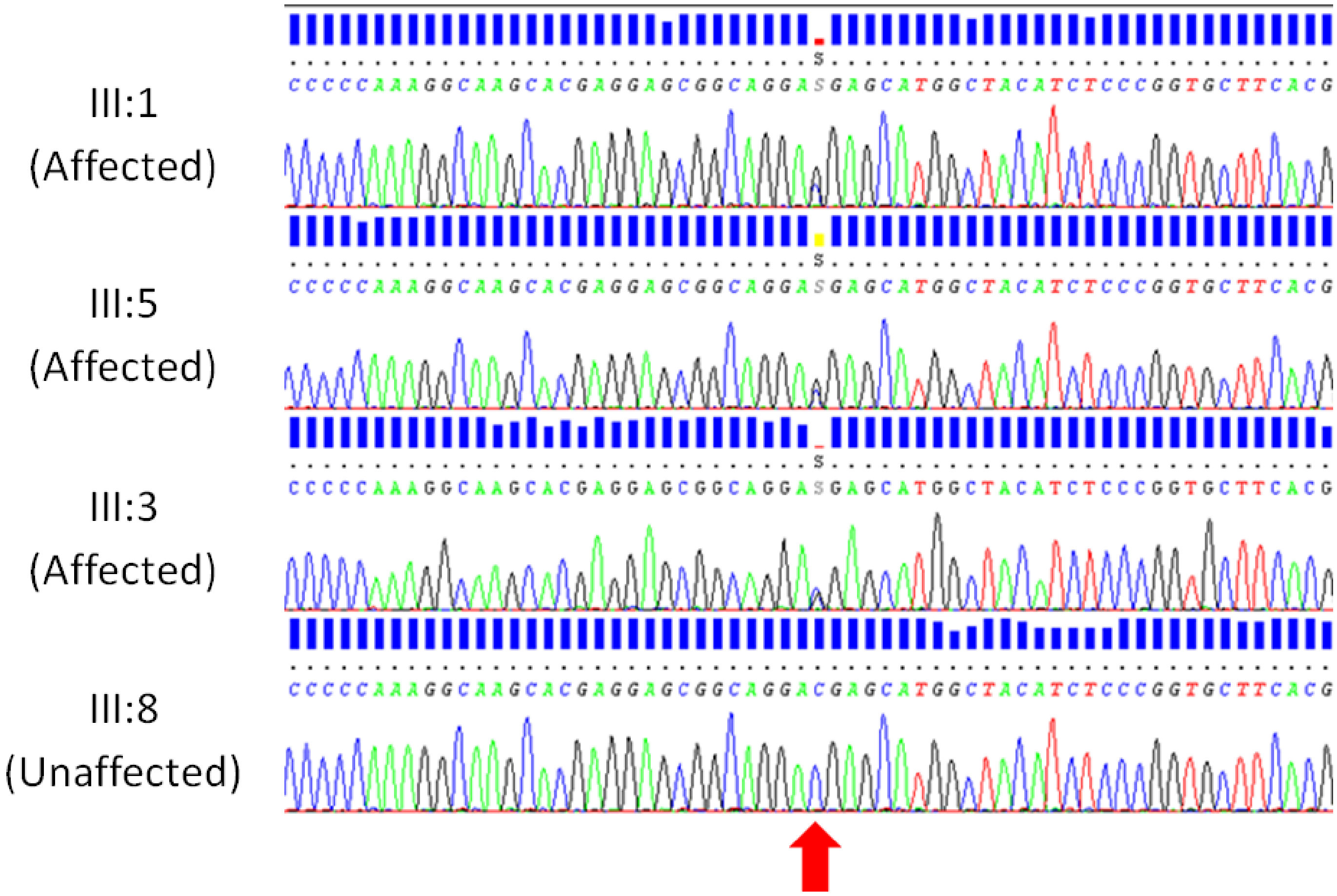

Supplementary References

1. Li H, Durbin R. Fast and accurate short read alignment with Burrows-Wheeler transform. Bioinformatics 2009;25(14):1754-1760.
2. Picard, ^^Broad Institute,, http://broadinstitute.github.io/picard/, ^2012
3. Koboldt DC, Chen K, Wylie T, et al. VarScan: variant detection in massively parallel sequencing of individual and pooled samples. Bioinformatics 2009;25(17):2283-2285.
4. Albers CA, Lunter G, MacArthur DG, McVean G, Ouwehand WH, Durbin R. Dindel: accurate indel calls from short-read data. Genome Res 2011;21(6):961-973.
5. Wang K, Li M, Hakonarson H. ANNOVAR: functional annotation of genetic variants from high-throughput sequencing data. Nucleic Acids Res 2010;38(16):e164.
6. dbSNP 135, ^^NCBI, www.ncbi.nlm.nih.gov/SNP/, ^2012
7. 1000 genomes, ^^NCBI, http://www.ncbi.nlm.nih.gov/variation/tools/1000genomes/, ^March 2012
8. Exome Variant Server, ^^NHLBI GO Exome Sequencing Project,,, http://evs.gs.washington.edu/EVS/, ^February 2012
9. Ensembl, http://www.ensembl.org, ^2012
10. Evgrafov OV, Mersiyanova I, Irobi J, et al. Mutant small heat-shock protein 27 causes axonal Charcot-Marie-Tooth disease and distal hereditary motor neuropathy. Nature genetics 2004; 36(6): 602-606.
11. Tang B, Liu X, Zhao G, et al. Mutation analysis of the small heat shock protein 27 gene in chinese patients with Charcot-Marie-Tooth disease. Archives of neurology 2005; 62(8): 1201-1207.
12. Kijima K, Numakura C, Goto T, et al. Small heat shock protein 27 mutation in a Japanese patient with distal hereditary motor neuropathy. Journal of human genetics 2005; 50(9): 473-476.
13. James PA, Rankin J, Talbot K. Asymmetrical late onset motor neuropathy associated with a novel mutation in the small heat shock protein HSPB1 (HSP27). Journal of neurology, neurosurgery, and psychiatry 2008; 79(4): 461-463.
14. Houlden H, Laura M, Wavrant-De Vrieze F, Blake J, Wood N, Reilly MM. Mutations in the HSP27 (HSPB1) gene cause dominant, recessive, and sporadic distal HMN/CMT type 2. Neurology 2008; 71(21): 1660-1668.
15. Dierick I, Baets J, Irobi J, et al. Relative contribution of mutations in genes for autosomal dominant distal hereditary motor neuropathies: a genotype-phenotype correlation study. Brain : a journal of neurology 2008; 131(Pt 5): 1217-1227.
16. Ikeda Y, Abe A, Ishida C, Takahashi K, Hayasaka K, Yamada M. A clinical phenotype of distal hereditary motor neuronopathy type II with a novel HSPB1 mutation. Journal of the neurological sciences 2009; 277(1-2): 9-12.

17. Luigetti M, Fabrizi GM, Madia F, et al. A novel HSPB1 mutation in an Italian patient with CMT2/dHMN phenotype. Journal of the neurological sciences 2010; 298(1-2): 114-117.
18. Mandich P, Grandis M, Varese A, et al. Severe neuropathy after diphtheria-tetanus-pertussis vaccination in a child carrying a novel frame-shift mutation in the small heat-shock protein 27 gene. Journal of child neurology 2010; 25(1): 107-109.
19. Capponi S, Geroldi A, Fossa P, et al. HSPB1 and HSPB8 in inherited neuropathies: study of an Italian cohort of dHMN and CMT2 patients. Journal of the peripheral nervous system : JPNS 2011; 16(4): 287-294.
